# Analysis of ex vivo drug response data of *Plasmodium* clinical isolates: the pros and cons of different computer programs and online platforms

**DOI:** 10.1186/s12936-016-1173-1

**Published:** 2016-03-02

**Authors:** Grennady Wirjanata, Irene Handayuni, Sophie G. Zaloumis, Ferryanto Chalfein, Pak Prayoga, Enny Kenangalem, Jeanne Rini Poespoprodjo, Rintis Noviyanti, Julie A. Simpson, Ric N. Price, Jutta Marfurt

**Affiliations:** Global and Tropical Health Division, Menzies School of Health Research, Charles Darwin University, PO Box 41096, Casuarina, Darwin, NT 0811 Australia; Centre for Epidemiology and Biostatistics, Melbourne School of Population and Global Health, University of Melbourne, Melbourne, Australia; Papuan Health and Community Development Foundation (PHCDF), Timika, Papua Indonesia; District Health Authority, Timika, Papua Indonesia; Department of Paediatrics, Faculty of Medicine, Gadjah Mada University, Yogyakarta, Indonesia; Eijkman Institute for Molecular Biology, Jl. Diponegoro 69, 10430 Jakarta, Indonesia; Centre for Tropical Medicine and Global Health, Nuffield Department of Clinical Medicine, University of Oxford, Oxford, UK

## Abstract

**Background:**

In vitro drug susceptibility testing of malaria parasites remains an important component of surveillance for anti-malarial drug resistance. The half-maximal inhibition of growth (IC_50_) is the most commonly reported parameter expressing drug susceptibility, derived by a variety of statistical approaches, each with its own advantages and disadvantages.

**Methods:**

In this study, licensed computer programs WinNonlin and GraphPad Prism 6.0, and the open access programs HN-NonLin, Antimalarial ICEstimator (ICE), and In Vitro Analysis and Reporting Tool (IVART) were tested for their ease of use and ability to estimate reliable IC_50_ values from raw drug response data from 31 *Plasmodium falciparum* and 29 *P. vivax* clinical isolates tested with five anti-malarial agents: chloroquine, amodiaquine, piperaquine, mefloquine, and artesunate.

**Results:**

The IC_50_ and slope estimates were similar across all statistical packages for all drugs tested in both species. There was good correlation of results derived from alternative statistical programs and non-linear mixed-effects modelling (NONMEM) which models all isolate data simultaneously. The user-friendliness varied between packages. While HN-NonLin and IVART allow users to enter the data in 96-well format, IVART and GraphPad Prism 6.0 are capable to analyse multiple isolates and drugs in parallel. WinNonlin, GraphPad Prism 6.0, IVART, and ICE provide alerts for non-fitting data and incorrect data entry, facilitating data interpretation. Data analysis using WinNonlin or ICE took the longest computationally, whilst the offline ability of GraphPad Prism 6.0 to analyse multiple isolates and drugs simultaneously made it the fastest among the programs tested.

**Conclusion:**

IC_50_ estimates obtained from the programs tested were comparable. In view of processing time and ease of analysis, GraphPad Prism 6.0 or IVART are best suited for routine and large-scale drug susceptibility testing.

**Electronic supplementary material:**

The online version of this article (doi:10.1186/s12936-016-1173-1) contains supplementary material, which is available to authorized users.

## Background

Malaria remains a serious public health problem in endemic countries [1]. Efforts to control and eliminate malaria have failed repeatedly, often due to the spread of drug-resistant parasites and vectors. Resistance has emerged and spread to all currently available anti-malarials and reinforces the need for better surveillance strategies. In vitro assays for assessing anti-malarial drug susceptibility are an important part of monitoring drug resistance and investigation of novel anti-malarial compounds [2]. Although artemisinin-based combination therapy (ACT) has been implemented widely for the treatment of falciparum malaria and has proven to be beneficial, it is important to consider that resistance to one component of the therapy can be masked by a partner drug which retains high anti-malarial efficacy. In vitro assays also provide an opportunity to assess drug susceptibility of parasites to individual drugs, thereby allowing preventive measures to be taken before clinical treatment failure occurs. In addition, in vitro assays enable the measurement of drug sensitivity without the confounding effects of clinical efficacy such as host immunity and the pharmacokinetics of the drug [2–4].

A variety of assays are available to measure drug susceptibility in *Plasmodium falciparum*. In most, the parasites’ drug susceptibility is defined by measuring growth (i.e., schizont maturation) or replication (i.e., re-invasion assays) in the presence of varying concentrations of anti-malarial compounds. Although these assays are highly informative, the comparison of data between laboratories or field sites is often problematic as numerous variations exist between protocols, such as the initial parasitaemia, incubation time, culture haematocrit, and the use of alternative media and supplements [5, 6].

Standard analysis of in vitro (culture-adapted *Plasmodium* strains) and ex vivo (fresh *Plasmodium* clinical isolates) assay data is commonly conducted by using non-linear regression to fit a sigmoid *E*_max_ model to each sample’s concentration-effect data. The sigmoid *E*_max_ model is comprised of four parameters: minimum and maximum effects, steepness (=slope) of the dose–response curve, and concentration of the drug required to inhibit growth to 50 % of that observed in the absence of the drug (IC_50_). In this approach, the definition of IC_50_ is based on the assumptions that there is: (a) a monotonic relationship between the dose of the compound tested and the response in the assay; and, (b) a consistent 50 % response can be defined clearly [7]. IC_50_ values can also be estimated using alternative statistical models, such as polynomial regression [8] and sigmoid inhibition models based on non-linear regression [9, 10]. Determination of reproducible IC_50_ values remains a challenge for investigators as it is time-consuming and often subjective as the process itself involves visual inspection of individual dose–response curves [10]. Moreover, laboratories have different criteria for accepting or rejecting assay data, leading to a variety of selection biases, such as excluding assays from highly resistant parasites for which IC_50_ estimates tend to be less precise [11].

Different computer programs and online platforms are available, each with its own algorithms and features, to facilitate the processing of raw drug assay data. In this study, five of the most commonly used analytical platforms were assessed by applying them to the same dataset of ex vivo drug response data collected from patients with clinical malaria and comparing the derived output parameters, their utility, robustness, and consistency of results.

## Methods

### Clinical isolates

From April 2012 to January 2013, *Plasmodium* species isolates were collected from patients with malaria attending an outpatient clinic in Timika, Papua Province, Indonesia. In this region, clinical trials have confirmed high levels of multidrug-resistant *P. vivax* and *P. falciparum* [12–14]. Patients with symptomatic malaria were recruited into the study if they had a single species infection with *P. falciparum* or *P. vivax,* with a parasitaemia of between 2000 and 80,000 μL^−1^ as determined by microscopy, and with synchronous infection with at least 70 % parasites at ring stage. Patients were excluded from the study if they had taken any anti-malarials in the preceding month. After written informed consent was obtained, 5 mL venous blood was collected by venipuncture. Host white blood cells (WBC) were removed using cellulose column filtration and packed infected red blood cells (IRBC) used for the ex vivo drug susceptibility assay.

### Ex vivo drug susceptibility assay

*Plasmodium* drug susceptibility was measured using a protocol modified from the World Health Organization (WHO) microtest as described previously [12, 15]. Two-hundred microlitres of a 2 % haematocrit blood medium mixture (BMM), consisting of RPMI 1640 medium plus 10 % AB+ human serum (*P. falciparum*) or McCoy’s 5A medium plus 20 % AB+ human serum (*P. vivax*) were added to each well of pre-dosed drug plates containing 11 serial concentrations (twofold dilutions) of the anti-malarials (maximum concentration shown in parentheses) chloroquine (2992 nM), piperaquine (769 nM), mefloquine (338 nM), artesunate (49 nM), and amodiaquine (158 nM). Pre-dosed drug plates were made by diluting the compounds in 50 % methanol, followed by lyophilization, and stored at 4 **°**C until use. All anti-malarials were obtained from the World Wide Antimalarial Resistance Network (WWARN) Malaria Drug Reference Material Scheme [16].

### Dose–response analysis

The raw dose–response data for each drug were visually checked to ensure that they were amenable to regression modelling. Five programs and/or online tools were evaluated.

### WinNonlin 4.1 (Pharsight Corporation)

IC_50_ values were estimated using the program WinNonlin 4.1 (Pharsight Corp) by fitting a sigmoid *E*_max_ model (Eq. 1) to each individual isolate’s concentration-effect data by non-linear regression

1 $$ E = E_{\hbox{max} } - \left( {E_{\hbox{max} } - E_{0} } \right) \times \left[ {\frac{{C^{\gamma } }}{{C^{\gamma } + IC_{50}^{\gamma } }}} \right].$$

*E* represents the percentage of schizonts observed after normalization to the control wells using the following equation:

$$ E = \frac{{E_{raw} }}{{E_{control} }} $$

*E*_*raw*_ is the percentage of schizont observed in the drug well and divided by the percentage of schizont observed in the control well (*E*_*control*_). Maximum inhibition occurs when there is no observable schizont (i.e., inhibition of growth), that is, *E* equals 0. In Eq. 1, *E*_*0*_ represents minimum per cent growth, *E*_max_ maximum per cent growth, *IC*_*50*_ the concentration of the drug required to inhibit 50 % of the control parasites’ schizont growth, *C* the drug concentration, and *γ* the steepness of the curve (slope).

Initial raw data were transposed into an Excel converter worksheet to fulfil the program’s template requirements. The converted data were then transposed into the program’s data table and analysed using the pharmacodynamic inhibitory sigmoid *E*_max_ model presented above (see Eq. 1). Key outputs and additional parameters are presented in Table 3. Warning messages were shown when potential inaccuracies were identified, such as steep response without observed values on the slope, and dose–response curves were provided for visual inspection.

Isolates with estimated *E*_max_ and E_0_ values above 15 % or below 100 and 0, respectively, were re-analysed. The majority of these results were caused by either resistant isolates in which the response was deemed inadequate at the highest drug concentration tested, or lower schizont counts at the lowest concentration tested than those of untreated controls. In such cases, additional extreme data points were added assuming E = 1 with no drug and 0 when very high drug concentrations were present.

### GraphPad Prism (version 6.01; GraphPad Software, Inc.)

Dose–response analysis of the log-transformed drug concentrations (represented by X in Eq. 2) and response (represented by Y in Eq. 2) was performed using the ‘log (inhibitor) *versus* response–variable slope’ equation (Hill equation):

2 $$ {\text{Y}} = \frac{{{\text{Bottom}} + \left( {{\text{Top}} - {\text{Bottom}}} \right)}}{{1 + \left( {\frac{{10^{{{\text{LogIC}}_{50} }} }}{{10^{\text{x}} }}} \right)^{{{\upgamma }}} }} $$

*Bottom* represents the value of *Y* for the minimal curve asymptote (response in the absence of drug), *Top* is the value of *Y* for the maximal curve asymptote (response produced by an infinitely high concentration of drug). *LogIC*_*50*_ is the logarithm of the drug concentration required to inhibit 50 % of growth, and *γ* represent the slope of the curve. The upper and lower limits of *γ* were constrained to 10 and 0, respectively.

Raw data were first transferred into an Excel converter worksheet to fulfil the program’s template requirements. The converted raw data were then transposed into the program’s Y-axis data table and the drug concentrations entered into the X-axis data table; the data were log-transformed prior to analysis. In GraphPad Prism 6.0, the results were available immediately after data entry. Key and additional outputs are outlined in Table 3. Dose–response curves were also produced for visual inspection. The program showed ‘ambiguous’ text warnings when it was unable to find the combination of parameters that best fit the data. A warning text was shown for the following reasons: (i) data were not collected over a wide enough range of drug concentrations; (ii) the model had redundant parameters (i.e., the model could be fitted with multiple sets of parameters); or, (iii) the model resulted in a biphasic curve, leading to different IC_50_ values [17]. In the case of ‘ambiguous’ results due to reason (i), additional extreme data points were added, assuming Y equals to 0 when very high drug concentrations were present, and data were re-analysed.

### HN-NonLin

HN-NonLin is a freeware program [18] that uses log-transformed data to fit a polynomial regression model with the following equation (Eq. 3):

3 $$ Y = A + Bx + Cx^{2} + Dx^{3} + Ex^{4} $$

The goal of polynomial regression is to find the values of the parameters *A*, *B*, *C*, etc., that are going to generate the best-fit curve for the observed data points. When using a polynomial regression model, investigators need to specify the order of the polynomial (i.e., the numbers of parameters to be fitted into the equation) [19]. In HN-NonLin, users can choose the order of the polynomial (i.e., 1–7) that will determine the shape of the dose–response curve: Polynomial ‘1’ will give a straight line, while polynomial ‘7’ will produce a perfect interpolation (i.e., the regression line will pass through all data points). In this study, polynomial ‘3’ was chosen since polynomials at higher order will produce perfect correlation, but undesirable oscillations caused by outliers [20].

To conduct data modelling using HN-NonLin, raw data were transferred into an Excel converter worksheet in order to fit the 96-well plate format of the program. After the polynomial was set, data modelling was performed and the key parameters, as well as additional outputs generated as presented in Table 3. As with the other programs, additional extreme data points were added in cases where drug-response data were deemed inadequate, assuming that no schizont was observed at very high drug concentrations.

### ICEstimator 1.2

The ICEstimator is a freeware online program that carries out a non-linear regression on the relative concentration-effect points by using an inhibitory sigmoid *E*_max_ model [9].

Raw data were transferred into an Excel converter worksheet to comply with the program’s requirements. Converted raw data were then entered via the ICEstimator 1.2 website [21]. For the analysis of each isolate, drug concentration (x-axis) and response data (y-axis) needed to be entered individually. In the current study, the unit for concentration was set to ‘nM’ and for response ‘other’ was chosen. In addition, ‘schizont counting’ was chosen as the method for analysis. Data modelling was performed online generating the outputs outlined in Table 3. Dose–response curves were available for individual inspection. Warning signs were displayed if: (i) the ratio of the upper and lower limits of the 95 % CI of the IC_50_ was greater than two; (ii) convergence was unsuccessful, even when the slope was set at 10; (iii) the parasite’s growth ratio (mean effect at drug-free control/mean effect at maximum drug concentration) was less than two; (iv) the lower limit of the IC_50_ 95 % CI was less than 0; and/or, (v) IC_99_> maximum concentration [9]. The results, as well as dose–response curves were displayed directly on the website and were available for download in portable data file (pdf) and comma-separated value (csv) format.

### In Vitro Analysis and Reporting Tool (IVART)

IVART is a freeware online program that was developed by WWARN. IVART is a program that is based on the code of the ICEstimator program and described in detail on the WWARN website [10].

Raw data were transferred into an Excel converter worksheet and converted into a 96-well format to comply with the program’s requirements. Converted raw data were then entered into the WWARN pre-build Excel worksheet as per the developer’s instructions [22]. This worksheet was then uploaded through the WWARN website [23] and data analysis performed online. The results took approximately 1 min to be generated following data upload and could be downloaded as pdf and/or csv files. Key and additional outputs are outlined in Table 3. In the pdf output, warning messages were shown when (a) drug concentration ranges were incorrect; or, (b) core criteria, as described elsewhere [10], were not met. If warning signs were shown, additional drug concentrations and data points were added as described above and the data re-analysed.

### Non-linear mixed-effects modelling using the package NONMEM

The estimates obtained by all the programs tested were compared with the estimates produced by NONMEM. NONMEM is a commercial software package that analyses data from all isolates simultaneously using non-linear mixed-effects models to take into account the two sources of variability, within- and between-isolate variability. NONMEM allows the specification of any non-linear regression equation to describe the effect-concentration curves and can incorporate other confounding factors of the drug assay, such as parasite stage composition at the start of the assay, assay duration, and the use of different drug plate batches. For this analysis, the sigmoid *E*_max_ model (i.e., Eq. 1) was used and no additional confounding factors were included. The derivation of IC_50_ values using NONMEM is described in detail elsewhere [24].

### Data analysis

Results from each program were pooled and statistical analysis conducted using GraphPad Prism 6.0. Geometric mean and 95 % reference range of IC_50_ and slope parameters were calculated for each drug using all available programs. NONMEM was used as the reference method and parameter estimates compared with each of the other methods. Bland–Altman plots using the derived IC_50_ estimates were created to compare agreement between programs.

In addition, a qualitative analysis was conducted to assess the ease of use and application of each methodology. This included how the raw data were processed, data conversion and/or transformation, data upload, data analysis, processing time, capability to perform batch analysis, capability of offline analysis, and costs.

### Ethical approval

Ethical approval for this project was obtained from the Human Research Ethics Committee of the Northern Territory Department of Health and Families and Menzies School of Health Research (HREC 2010–1396), Darwin, Australia, and the Eijkman Institute Research Ethics Commission (EIREC-47), Jakarta, Indonesia.

## Results

### Overview and comparison of outputs

**Fig. 1:**
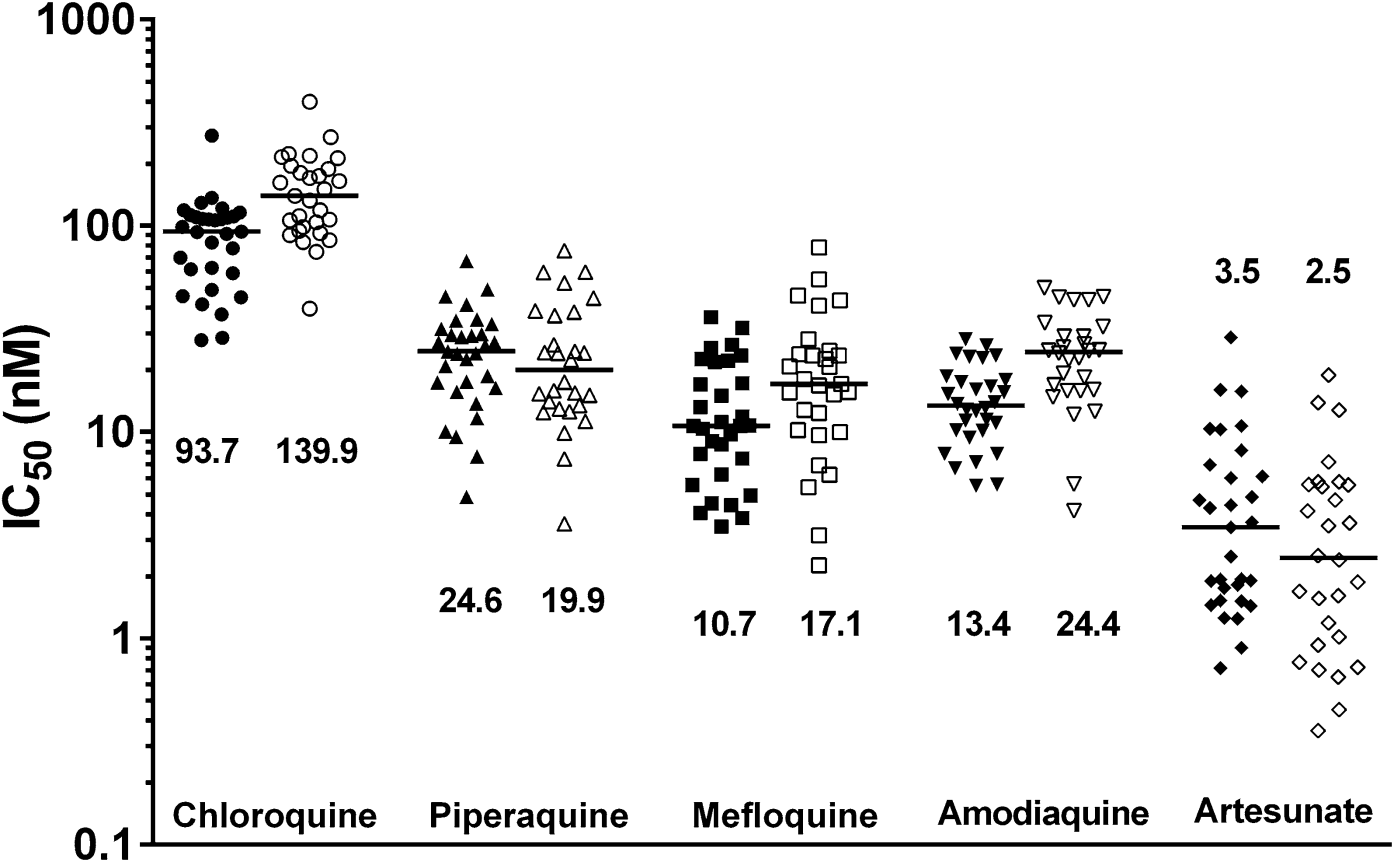
Ex vivo drug susceptibility (median IC_50_s) of standard anti-malarials in *Plasmodium falciparum* (*closed symbols*) and *Plasmodium vivax* (*open symbols*) clinical field isolates. IC_50_ estimates depicted in the Figure were derived by using NONMEM

Ex vivo drug susceptibility assays to chloroquine, amodiaquine, piperaquine, mefloquine, and artesunate were assessed in field isolates from 60 patients presenting with single-species infections of *P. falciparum* (n = 31) and *P. vivax* (n = 29). Drug susceptibility data were successfully derived from all samples. Baseline characteristics of the isolates processed are presented in Table 1 and the derived drug susceptibility for both species depicted in Fig. 1. The population geometric mean IC_50_s were similar for the five different methodologies with no significant differences when compared to the NONMEM reference estimates (see Tables 2 and 4). With the exception of HN-NonLin, all programs generated estimates of the slope (*γ*) and corresponding 95 % confidence intervals (Table 3).

**Table 1:** Baseline characteristics of isolates used for analysis

Baseline characteristic	*P. falciparum* (n = 31)	*P. vivax* (n = 29)
Median (range) delay from venipuncture to start of culture (minutes)	116 (80–180)	131 (75–210)
Median (range) duration of assay (hours)	45 (42–50)	46 (32–50)
Geometric mean (95 % CI) parasitaemia (asexual parasites/μL)	11,832 (9282–15,084)	17,233 (11,902–24,952)
Median initial % (range) of parasites at ring stage	100 (100–100)	95 (70–99)
Mean (95 % CI) schizont count at harvest	43 (36–49)	44 (40–47)

CI, confidence interval

**Table 2:** Comparison of IC_50_ values for concentration-effect curves of anti-malarial drugs against *Plasmodium falciparum* and *Plasmodium vivax* generated by different programs

	*P. falciparum*				*P. vivax*		
	Method	Geometric mean IC_50_ [nM] (95 % CI)	Median difference IC_50_^a^ [nM] (range)		Method	Geometric mean IC_50_ [nM] (95 % CI)	Median difference IC_50_^a^ [nM] (range)
Chloroquine (n = 31)	*NONMEM*	*91.40 (74.39–108.40)*		Chloroquine (n = 29)	*NONMEM*	*151.9 (124.2–179.6)*	
	GP	81.43 (67.67–98.00)	0.42 (−7.80 to 6.50)		GP	137.8 (115.8–163.9)	0.37 (−50.0-32.0)
	HNL	81.59 (67.26–98.97)	0.42 (−5.50 to 13.0)		HNL	136.7 (114.3–163.5)	0.33 (−47.0 to 35.0)
	ICE	82.59 (68.45–99.65)	1.50 (−5.00 to 11.0)		ICE	137.4 (115.7–163.2)	1.80 (−62.0 to 10.0)
	IVART	83.85 (69.20–101.6)	0.93 (−12.0 to 40.0)		IVART	135.2 (113.0–161.7)	1.90 (−62.0 to 17.0)
	WNL	81.40 (67.62–98.00)	0.51 (−5.40 to 5.10)		WNL	136.4 (114.7–162.1)	0.18 (−63.0 to 12.0)
Piperaquine (n = 31)	*NONMEM*	*25.65 (20.79–30.50)*		Piperaquine (n = 28)	*NONMEM*	*26.03 (18.99–33.08)*	
	GP	21.77 (17.79–26.64)	0.56 (−9.60 to 3.30)		GP	20.46 (15.51–26.99)	−0.08 (−3.80 to 2.30)
	HNL	22.35 (18.30–27.30)	−0.22 (−5.10 to 13.0)		HNL	21.26 (16.21–27.89)	0.68 (−4.80 to 6.90)
	ICE	22.72 (18.44–27.99)	0.44 (−3.00 to 4.90)		ICE	20.99 (15.99–27.54)	0.14 (−1.70 to 3.10)
	IVART	22.10 (17.88–27.32)	−0.02 (−11.0 to 4.90)		IVART	21.34 (16.35–27.85)	0.24 (−2.20 to 3.80)
	WNL	21.93 (17.92–26.85)	−0.28 (−9.20 to 3.30)		WNL	20.64 (15.68–27.18)	−0.05 (−1.80 to 1.70)
Mefloquine (n = 31)	*NONMEM*	*13.49 (10.26–16.71)*		Mefloquine (n = 29)	*NONMEM*	*21.67 (15.23–28.11)*	
	GP	10.99 (8.55–14.11)	0.01 (−1.30 to 2.90)		GP	16.53 (12.12–22.56)	0.27 (−0.81 to 4.40)
	HNL	11.31 (8.85–14.45)	0.24 (−0.89 to 3.80)		HNL	16.61 (12.24–22.54)	0.26 (−2.30 to 3.40)
	ICE	11.31 (8.84–14.46)	0.38 (−0.15 to 2.80)		ICE	16.82 (12.38–22.87)	0.56 (−0.32 to 3.20)
	IVART	10.59 (8.14–13.76)	0.03 (−5.10 to 3.10)		IVART	16.61 (12.00–23.00)	0.51 (−2.50 to 5.40)
	WNL	11.04 (8.59–14.17)	0.03 (−0.82 to 2.30)		WNL	16,341 (12.01–22.43)	0.12 (−1.20 to 3.00)
Amodiaquine (n = 30)	*NONMEM*	*14.53 (12.18–16.88)*		Amodiaquine (n = 28)	*NONMEM*	*24.97 (20.29–29.64)*	
	GP	13.40 (11.21–16.03)	−0.05 (−2.70 to 11.0)		GP	22.03 (17.4–27.80)	0.04 (−3.60 to 8.00)
	HNL	13.59 (11.42–16.18)	0.46 (−2.80 to 2.60)		HNL	22.31 (17.66–28.18)	1.10 (−2.10 to 4.50)
	ICE	13.24 (11.16–15.71)	0.04 (−1.10 to 0.87)		ICE	21.74 (17.34–27.25)	0.07 (−1.80 to 0.81)
	IVART	13.24 (11.16–15.71)	0.04 (−1.10 to 0.87)		IVART	21.88 (17.31–27.10)	−0.03 (−1.80 to 0.81)
	WNL	13.38 (11.22–15.96)	−0.04 (−1.30 to 11.0)		WNL	21.88 (17.36–27.57)	−0.02 (−2.20 to 4.30)
Artesunate (n = 31)	*NONMEM*	*5.43 (3.23–7.63)*		Artesunate (n = 28)	*NONMEM*	*4.12 (2.38 to 5.85)*	
	GP	3.59 (2.48–5.19)	0.01 (−0.14 to 1.10)		GP	2.47 (1.63–3.75)	0.01 (−0.64 to 0.82)
	HNL	3.57 (2.52–5.07)	−0.01 (−2.60 to 11.0)		HNL	2.55 (1.69–3.85)	0.06 (−0.73 to 2.60)
	ICE	3.51 (2.48–4.67)	0.05 (−0.14 to 0.99)		ICE	2.50 (1.65–3.79)	0.04 (−0.30 to 0.79)
	IVART	3.54 (2.54–4.94)	0.07 (−2.90 to 0.84)		IVART	2.45 (1.61–3.72)	0.01 (−0.63 to 0.43)
	WNL	3.61 (2.49–5.23)	0.01 (−0.40 to 10.0)		WNL	2.48 (1.63–3.76)	0.01 (−0.41 to 1.00)

^a^Median difference (range) for each method compared with NONMEM (values in italics) used as the reference method for comparison

**Table 3:** Key outputs and features of the different analysis tools

	**Method**					
	**NONMEM**	**WinNonlin**	**GraphPad Prism 6.0**	**HN-Nonlin**	**ICEstimator 1.2**	**IVART**
Ease of use						
Free				✔	✔	✔
96-well format^a^				✔		✔
Batch analysis of multiple drugs		✔	✔	✔		✔
Batch analysis of multiple isolates	✔^e^	✔	✔			✔
Offline processing		✔	✔	✔		
Key output						
IC_50_ estimate	✔	✔	✔	✔	✔	✔
Slope estimate	✔	✔	✔		✔	✔
Confidence interval of estimates	✔	✔	✔		✔	✔
Success criteria and warning signs		✔	✔		✔	✔
Dose–response curve		✔	✔	✔	✔	✔
Data processing and analysis						
Data conversion		Critical	Critical	Easy	Critical	Easy
Approximate time needed for data conversion (seconds)^b, c^		45	25	25	25	25
Data upload/entry		Critical	Critical	Easy	Critical	Easy
Approximate time needed for data upload and processing (seconds)^b, d^		120	15	20	300	90
Approximate total time needed to obtain estimates (seconds)		165	40	45	325	115

^a^Based on the capability to enter the data in 96-well format

^b^Based on the capability to run one isolate with five different drugs in duplicates distributed on two different 96-well plates

^c^Time measurement includes the time needed to transfer raw data into a ‘converter’ spreadsheet and double checking the converted data to ensure that raw data was correctly entered and converted

^d^Time measurement includes the time needed to transfer the converted raw data into the corresponding programs until the outputs were successfully transferred into a separate analysis spreadsheet

^e^For NONMEM, data from all isolates are modelled simultaneously

For resistant isolates, both the IC_50_ and the shape of the dose response curve shift, and hence both parameters, are required to calculate the inhibitory responses at other concentrations, such as the IC_90_ or IC_99_. All programs produced dose–response curves for visual inspection, but only WinNonlin, GraphPad Prism 6.0, and HN-NonLin generated statistics for the goodness of fit of the final model. Although this statistic is not shown in ICE and IVART, warning messages are displayed when the regression fails to produce an acceptable fit.

Visual inspection of both raw data and dose-response curves is inevitable for final interpretation of IC_50_ estimates. When data cannot be modelled for reasons such as similar schizont growth at each drug concentration compared to the drug-free control, or non-sigmoidal curves, none of the software packages generated IC_50_ values or drug response curves. In these cases, ICE, IVART and WinNonlin also displayed error messages. For assays with a large discrepancy of response between duplicate concentrations on the slope, GraphPad Prism 6.0 and WinNonlin tended to produce higher IC_50_ estimates and lower slope values compared to the other packages. This shift in IC_50_s was most prominent when one of duplicates was zero on the slope (i.e., GraphPad Prism 6.0 and WinNonlin automatically excluded the zero value as outlier).

The IVART, as described by Woodrow and colleagues [10], was designed primarily for ≥48 h in vitro re-invasion assays, rather than the schizont maturation assay used in the current analysis. IVART generates a growth ratio, defined as the uninhibited growth divided by maximally inhibited growth. In the schizont maturation assay, maximum inhibition leads to zero schizont count, thus producing infinite values at these concentrations [10]. Nevertheless, IVART could still be successfully applied to the schizont maturation data, producing similar estimates as the other programs.

### Agreement with NONMEM

Bland–Altman plots were produced to measure the agreement between the different programs and NONMEM for every anti-malarial according to the species of infection. Overall, there was good concordance between all programs and NONMEM. The biases (mean differences in estimates) were close to zero for all drugs assessed (range 3.80–1.76 nM; Additional files 1, 2, 3, 4 and 5). In total, 80 out of 1485 (5.4 %) assays had differences outside the 95 % limits of agreement of the mean difference and were termed outliers. The majority of these outliers (30/80, 37.5 %) were derived from assays with a large discrepancy in duplicate microscopy readings, possibly caused by human error, leading to responses with biphasic curves [9, 25]. In these outliers, the difference in IC_50_ values with the NONMEM value was higher in GraphPad Prism 6.0 compared to other methods. Data with a very steep slope (i.e., slope estimate = 10) accounted for 30 % (24/80) of outliers. For all these outliers, NONMEM consistently produced lower slope values compared to other methods, ranging from 2.9 to 4.9 (Table 4). For the assays with steep slopes, GraphPad Prism 6.0, IVART and ICE tended to produce higher IC_50_ estimates than NONMEM. In total, 23.8 % (19/80) of the outliers had parasite growth at the highest concentration of the drug tested (i.e., 100 % inhibition was never reached). Whereas NONMEM interpreted these data within the context of the whole population, for the other programs, *E*_min_ was anchored to zero, assuming zero growth was eventually reached with very high drug concentrations. However, this approach needs to be interpreted with caution. Data with ‘plateau responses’ (i.e., the same schizont counts were recorded for two or more different drug concentrations before it reached the maximum effect) were present in 5 % (4/80) assay outliers. The rest of the outliers (3.7 %; 3/80) were generated by IVART where a very wide confidence interval for the IC_50_ values was generated.

**Table 4:** Comparison of slope values for concentration-effect curves of anti-malarial drugs against *Plasmodium falciparum* and *Plasmodium vivax*

	*P. falciparum*				*P. vivax*		
	Method	Geometric mean slope (95 % CI)	Median difference slope^a^ (range)		Method	Geometric mean slope (95 % CI)	Median difference slope^a^ (range)
Chloroquine (n = 31)	*NONMEM*	*3.09 (2.72–3.46)*		Chloroquine (n = 29)	*NONMEM*	*3.86 (2.99–4.72)*	
	GP	3.15 (2.66–3.73)	0.03 (−0.20 to 4.85)		GP	3.48 (2.75–4.39)	0.06 (−0.46 to 2.82)
	ICE	3.33 (2.84–3.91)	0.17 (0.03 to 4.85)		ICE	3.67 (2.93–4.60)	0.20 (−0.46 to 2.82)
	IVART	3.40 (2.91–3.97)	0.23 (−1.67 to 4.69)		IVART	3.55 (2.85–4.42)	0.21 (−2.20 to 4.90)
	WNL	3.08 (2.64–3.59)	0.01 (−0.18 to 2.88)		WNL	3.43 (2.71–4.35)	0.03 (−0.46 to 2.82)
Piperaquine (n = 31)	*NONMEM*	*3.17 (2.58–3.76)*		Piperaquine (n = 28)	*NONMEM*	*3.80 (3.17–4.43)*	
	GP	3.13 (2.54–3.85)	0.08 (−0.50 to 3.63)		GP	3.88 (3.03–4.96)	0.02 (0.29 to 4.32)
	ICE	3.21 (2.59–3.96)	0.21 (−0.07 to 3.63)		ICE	4.13 (3.26–5.25)	0.26 (0.03 to 4.32)
	IVART	3.07 (2.47–3.82)	0.20 (−2.59 to 4.60)		IVART	4.06 (3.24–5.09)	0.26 (−0.92 to 4.32)
	WNL	2.83 (2.37–3.37)	0.01 (−0.52 to 3.61)		WNL	3.82 (3.01–4.86)	−0.01 (−0.26 to 4.16)
Mefloquine (n = 31)	*NONMEM*	*2.48 (2.18–2.78)*		Mefloquine (n = 29)	*NONMEM*	*2.96 (2.53–3.38)*	
	GP	2.46 (2.10–2.89)	0.03 (−0.15 to 5.04)		GP	3.11 (2.48–3.91)	0.01 (−0.23 to 5.07)
	ICE	2.60 (2.24–3.02)	0.19 (0.01 to 5.04)		ICE	3.25 (2.64–4.00)	0.18 (0.04 to 5.19)
	IVART	2.38 (2.05–2.75)	0.08 (−0.86 to 0.92)		IVART	3.04 (2.53–3.65)	0.19 (−1.63 to 5.19)
	WNL	2.42 (2.08–2.83)	0.02 (−0.15 to 4.21)		WNL	3.02 (2.41–3.78)	−0.01 (−0.69 to 5.05)
Amodiaquine (n = 30)	*NONMEM*	*4.43 (3.37–5.50)*		Amodiaquine (n = 28)	*NONMEM*	*3.95 (3.26–4.65)*	
	GP	3.68 (2.89–4.70)	0.06 (−0.37 to 4.67)		GP	3.89 (3.03–4.98)	0.06 (−0.54 to 4.50)
	ICE	4.01 (3.22–5.00)	0.24 (0.03 to 4.67)		ICE	4.12 (3.28–5.18)	0.19 (−0.24 to 4.50)
	IVART	4.01 (3.22–5.00)	0.24 (0.03 to 4.67)		IVART	4.17 (3.33–5.24)	0.19 (−0.24 to 4.50)
	WNL	3.64 (2.86–4.63)	0.05 (−0.43 to 3.67)		WNL	3.87 (3.03–4.95)	0.02 (0.28 to 4.47)
Artesunate (n = 31)	*NONMEM*	*2.74 (2.38–3.10)*		Artesunate (n = 28)	*NONMEM*	*2.74 (2.38–3.10)*	
	GP	2.63 (2.19–3.15)	−0.05 (−0.35 to 4.55)		GP	3.67 (2.75–4.89)	0.18 (−0.54 to 4.79)
	ICE	2.94 (2.49–3.46)	0.20 (0.35 to 4.55)		ICE	3.92 (3.01–5.10)	0.24 (0.01 to 4.79)
	IVART	3.03 (2.52–3.65)	0.32 (−0.68 to 7.12)		IVART	3.84 (2.94–5.01)	0.18 (−0.38 to 4.38)
	WNL	2.63 (2.19–3.15)	−0.01 (−0.37 to 4.51)		WNL	3.64 (2.76–4.79)	0.14 (−0.31 to 4.49)

^a^Median difference (range) for each method compared with NONMEM (values in italics) used as the reference method for comparison

### Qualitative analysis

The key features of each package are summarized in Table 3.

#### Data conversion and transformation

All of the programs had individual template requirements. To fulfil these, raw data were converted into an Excel file first before transposing into the appropriate template. This process was critical and error-prone for WinNonlin, ICE and GraphPad Prism 6.0, where raw data needed to be entered manually in either row or column format. Data conversion for HN-NonLin and IVART was easier, since the data could be transferred in a 96-well format, reducing the risk for transcription errors. The in vitro assay relies on a process of normalizing the response at each concentration against a control well with no drug exposure. With the exception of WinNonlin, the normalization algorithm is included in the analysis for all the statistical packages assessed in this study.

#### Plate format and templates

Drug susceptibility assays generally use drug plates in a 96-well format. Although the eight-well rows of a 96-well plate are sufficient to generate valid dose–response curves and IC_50_ estimates [4], most investigators prefer to use the 12-well columns of a 96-well plate in order to have a broader drug concentration range. In WinNonlin, GraphPad Prism 6.0, HN-NonLin, and IVART, users can enter pre-made drug concentration templates prior to processing, without the need to reset the template for every single analysis. This feature also allows the analysis of multiple drugs for the same isolate. This is not possible with ICE, which requires the drug concentration and response to be entered manually for each drug per isolate.

The number of drugs that can be assessed also varies. GraphPad Prism 6.0 allows the user to enter a user-defined number of drug concentrations as long the drug concentrations entered into the program are log-transformed. The drug template in HN-NonLin allows a maximum of 12 different drugs with seven different concentrations and one control well in a 96-well format. In IVART, the layout is more flexible and allows testing of eight to 12 different drugs with seven different concentrations plus one control well per drug, or 11 different concentrations and one control well per drug. Different 96-well plate templates employed by these programs can be suited to accommodate the investigator’s needs. With horizontal drug dilutions, investigators are able to obtain more data points and a wider dynamic range with a specific drug; this is particularly useful when testing novel anti-malarials with unknown IC_50_s. With vertical dilutions, investigators are able to test more anti-malarials in parallel, but with a lower dynamic range, an approach more suited for anti-malarials for which the IC_50_ range is well defined.

#### High throughput analysis

The ability to perform multiple plate analysis provides significant advantages for high throughput processing of large numbers of samples and a range of different anti-malarials. This feature is only available for IVART and GraphPad Prism 6.0.

#### Data processing time

Outputs from all programs can be obtained as an Excel worksheet (HN-NonLin), a csv file (ICE and IVART), pdf file (IVART), or can be exported to Word or Excel (WinNonlin and GraphPad Prism 6.0). The process from raw data conversion until final outputs was timed for each package. Since some programs are capable of analysing multiple drug plates and/or isolates, timing was performed by running a single isolate for five drugs on two 96-well plates (=single run). A single run could be completed on average in 40 s with GraphPad Prism 6.0 since multiple drug plates can be analysed in parallel and the results are updated instantly when new data are entered. HN-NonLin took 45 s, with the delay attributable to data from different plates having to be entered separately. Results from IVART can be obtained in approximately 90 s because raw data need to be uploaded to, and results downloaded from a server. A single run for ICE took 4–5 min, the main reason being that response data need to be entered per drug and per isolate.

#### Offline capabilities

For investigators working in resource-limited field settings with unreliable internet connection, the capability of offline data analysis has significant advantages. IVART and ICE require internet connection for uploading raw data to a server and calculations are generated online. WinNonlin, GraphPad Prism 6.0 and HN-NonLin allow offline data analysis.

## Conclusions

The lack of standardized approaches to the analysis of in vitro drug susceptibility continues to confound anti-malarial resistance surveillance and comparison of data generated from different laboratories. Reassuringly, the current study demonstrates that the various statistical programs produce good overall concordance for IC_50_ estimates across different drugs and for both *Plasmodium* species tested. However, there were some important differences. In 5.4 % of drug assays, there were significant differences between approaches. In general, these were attributable to how the packages dealt with noisy data. Since serial dilutions of drug concentrations are almost universally adopted, the confidence of estimates for highly resistant isolates can be wide. No matter which analytical platform is used, these important isolates need to be individually scrutinized.

For routine and medium to high throughput drug susceptibility testing, GraphPad Prism 6.0 and IVART offered distinct advantages, including their capability to process multiple plates in parallel and the availability of all necessary output parameters to assess the accuracy of data modelling and hence, facilitate interpretation of the final data. For smaller scale drug testing, all statistical packages tested generated comparable IC_50_ estimates. Investigators working in remote areas with poorer infrastructure, including limited internet connection, may prefer the freely available HN-NonLin for data analysis.

## Additional files

10.1186/s12936-016-1173-1 Bland–Altman plots of agreement between NONMEM and ICEstimator 1.2 in *Plasmodium falciparum* (left) and *Plasmodium vivax* (right). Dotted lines indicate 95 % limits of agreement. Data for artesunate were slightly skewed and could bias the estimated mean difference and 95 % limits of agreement.
10.1186/s12936-016-1173-1 Bland–Altman plots of agreement between NONMEM and GraphPad Prism 6.0 in *Plasmodium falciparum* (left) and *Plasmodium vivax* (right). Dotted lines indicate 95 % limits of agreement. Data for artesunate were slightly skewed and could bias the estimated mean difference and 95 % limits of agreement.
10.1186/s12936-016-1173-1 Bland–Altman plots of agreement between NONMEM and HNNonlin in *Plasmodium falciparum* (left) and *Plasmodium vivax* (right). Dotted lines indicate 95 % limits of agreement. Data for artesunate were slightly skewed and could bias the estimated mean difference and 95 % limits of agreement.
10.1186/s12936-016-1173-1 Bland–Altman plots of agreement between NONMEM and IVART in *Plasmodium falciparum* (left) and *Plasmodium vivax* (right). Dotted lines indicate 95 % limits of agreement. Data for artesunate were slightly skewed and could bias the estimated mean difference and 95 % limits of agreement.
10.1186/s12936-016-1173-1 Bland–Altman plots of agreement between NONMEM and WinNonlin in *Plasmodium falciparum* (left) and *Plasmodium vivax* (right). Dotted lines indicate 95 % limits of agreement. Data for artesunate were slightly skewed and could bias the estimated mean difference and 95 % limits of agreement.

## Abbreviations

ACT: artemisinin-based combination therapy
BMM: blood medium mixture
csv: comma-separated value
IC_50_: half-maximal inhibition of growth
ICE: antimalarial ICEstimator
IRBC: infected red blood cell
IVART: in vitro analysis and reporting tool
NONMEM: non-linear mixed-effects modelling
pdf: portable data file
WBC: white blood cell
WHO: World Health Organization
WWARN: worldwide antimalarial resistance network
