# Supplementary figures and images for: Analysis of ex vivo drug response data of Plasmodium clinical isolates: the pros and cons of different computer programs and online platforms
Source: Malar J. 2016 Mar 2;15:137. doi: 10.1186/s12936-016-1173-1 (PMC4776429; doi:10.1186/s12936-016-1173-1)

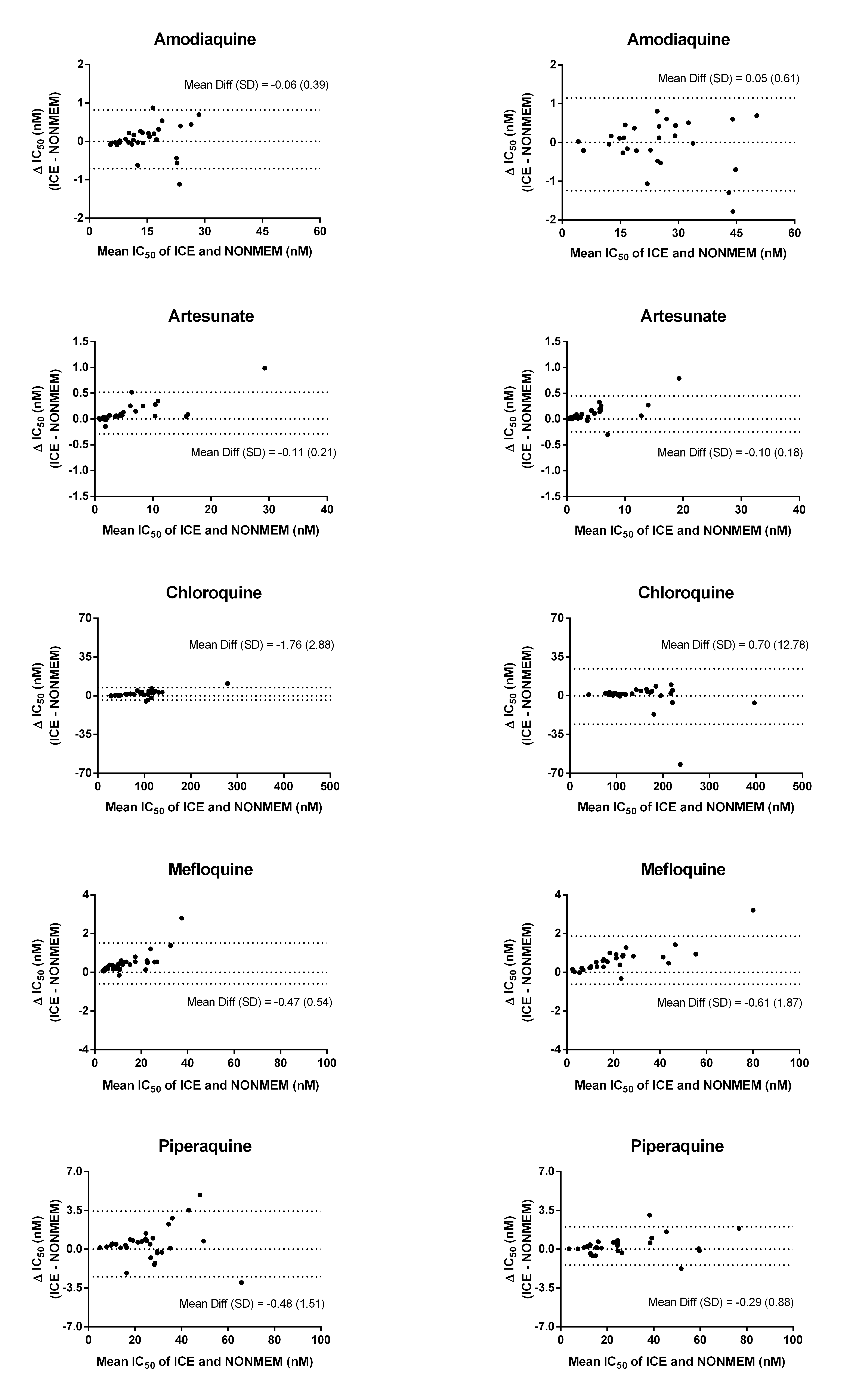

Supplement: Supplementary file 1 — 10.1186/s12936-016-1173-1 Bland–Altman plots of agreement between NONMEM and ICEstimator 1.2 in Plasmodium falciparum (left) and Plasmodium vivax (right). Dotted lines indicate 95 % limits of agreement. Data for artesunate were slightly skewed and could bias the estimated mean difference and 95 % limits of agreement. [file 12936_2016_1173_MOESM1_ESM.tif]

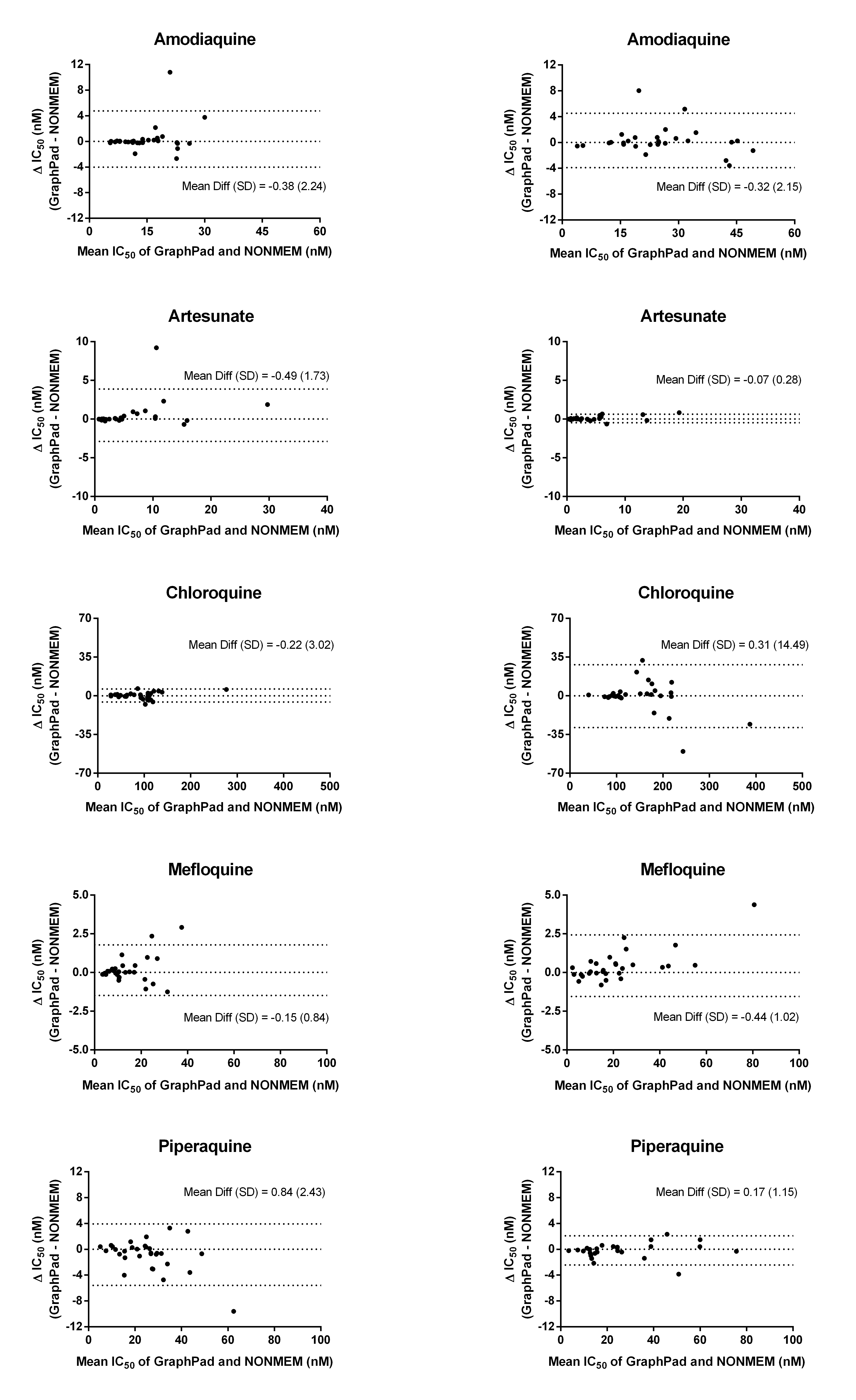

Supplement: Supplementary file 2 — 10.1186/s12936-016-1173-1 Bland–Altman plots of agreement between NONMEM and GraphPad Prism 6.0 in Plasmodium falciparum (left) and Plasmodium vivax (right). Dotted lines indicate 95 % limits of agreement. Data for artesunate were slightly skewed and could bias the estimated mean difference and 95 % limits of agreement. [file 12936_2016_1173_MOESM2_ESM.tif]

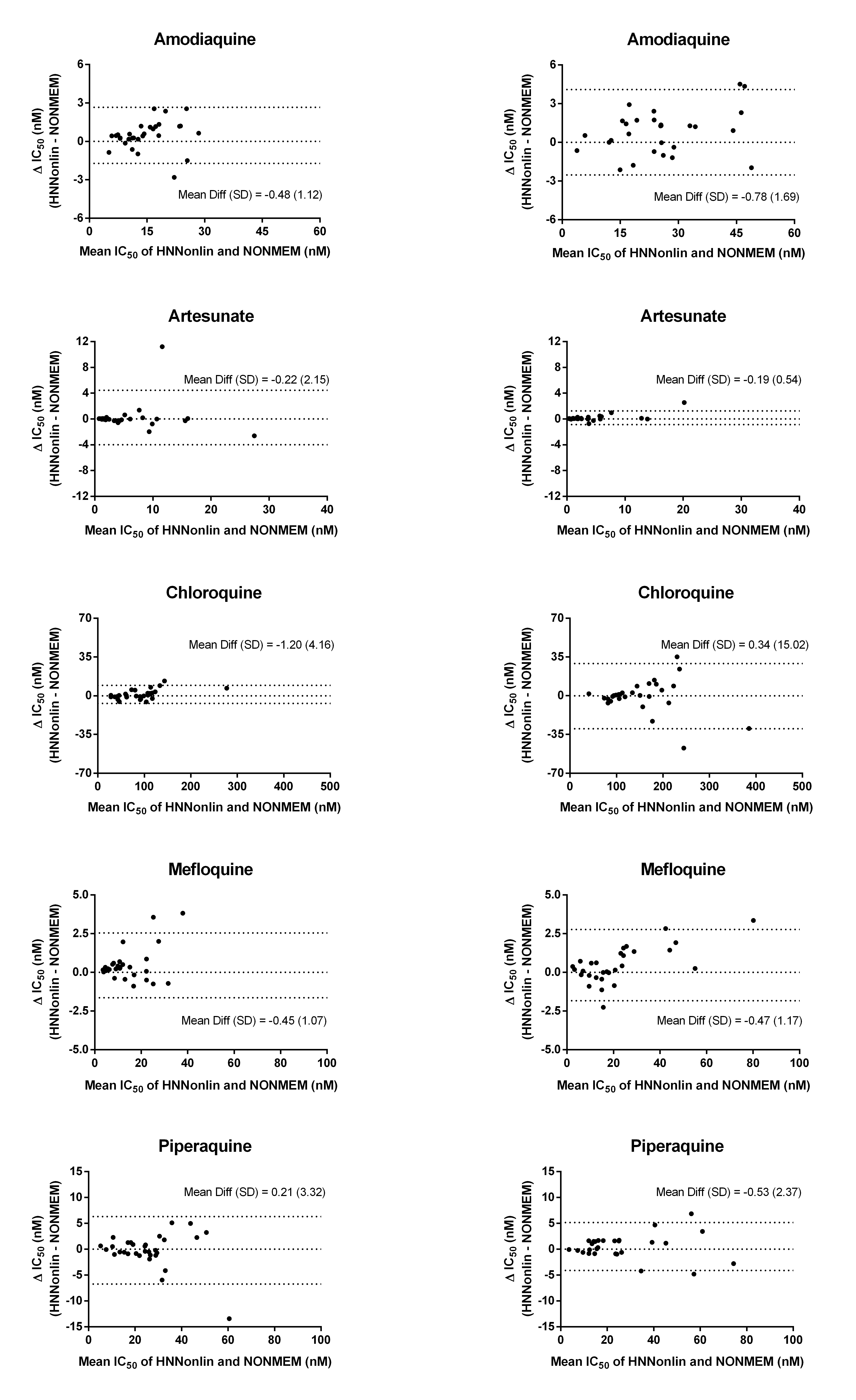

Supplement: Supplementary file 3 — 10.1186/s12936-016-1173-1 Bland–Altman plots of agreement between NONMEM and HNNonlin in Plasmodium falciparum (left) and Plasmodium vivax (right). Dotted lines indicate 95 % limits of agreement. Data for artesunate were slightly skewed and could bias the estimated mean difference and 95 % limits of agreement. [file 12936_2016_1173_MOESM3_ESM.tif]

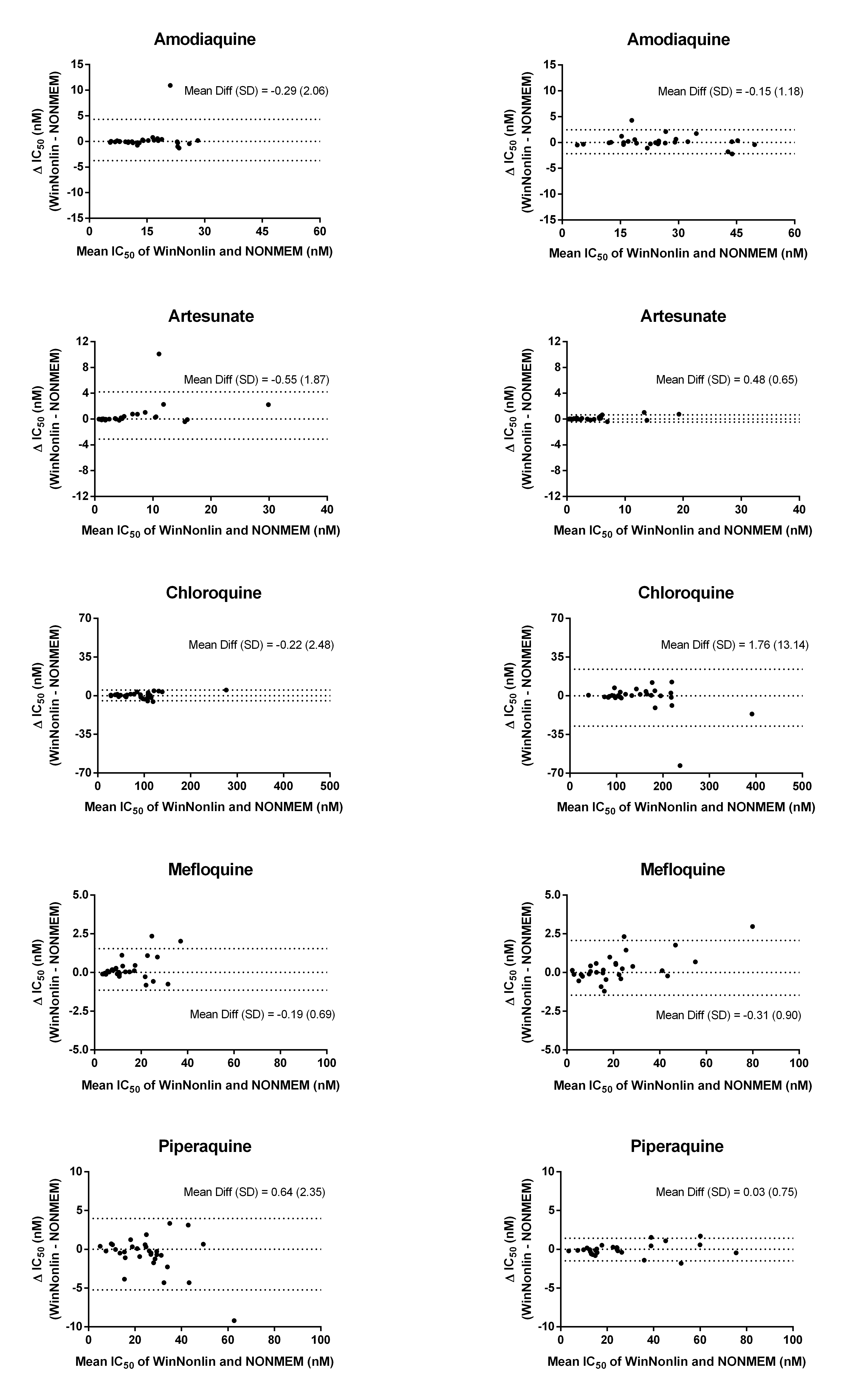

Supplement: Supplementary file 5 — 10.1186/s12936-016-1173-1 Bland–Altman plots of agreement between NONMEM and WinNonlin in Plasmodium falciparum (left) and Plasmodium vivax (right). Dotted lines indicate 95 % limits of agreement. Data for artesunate were slightly skewed and could bias the estimated mean difference and 95 % limits of agreement. [file 12936_2016_1173_MOESM5_ESM.tif]
